# Inter-Professional Education Interventions, and Practice Outcomes Related to Healthcare Setting and Patients Within Mental Healthcare: A Scoping Review

**DOI:** 10.5334/pme.1084

**Published:** 2024-02-20

**Authors:** Qian Hui Chew, Ethan Jian-Hui Maniam, Kang Sim

**Affiliations:** 1 Research Division, Institute of Mental Health, Singapore; 2 Yong Loo Lin School of Medicine, National University of Singapore, Singapore; 3 West Region, Institute of Mental Health, Singapore; 4 Lee Kong Chian School of Medicine, Nanyang Technological University, Singapore

## Abstract

**Introduction:**

This scoping review aimed to examine 1) types of inter-professional (IPE) interventions using Strosahl’s typology framework, 2) practice outcomes related to healthcare setting and patients using Kirkpatrick’s model of training evaluation, and 3) enablers and challenges related to the effectiveness of IPE interventions specific to the mental healthcare setting in order to guide the development of such future programs.

**Methods:**

This scoping review was conducted in accordance with the methodology of the Joanna Briggs Institute for scoping reviews. Several databases were searched for relevant studies from database inception until December 2023. Articles were included if it 1) involved IPE interventions within mental healthcare, 2) reported practice outcomes related to healthcare setting and patients, and 3) was published in English. Variables of interest included the mode of IPE intervention using Strosahl’s typology, enablers, and challenges related to IPE interventions.

**Results:**

Overall, 16 studies were included. IPE intervention outcomes within healthcare setting related to shifts in practice culture, engagement with family members, and increased collaborations with other inter-professional groups. Reported patient outcomes included clinical improvements (e.g., reduced depression and anxiety, psychotropic drug use, better psychosocial functioning), patient empowerment, satisfaction, and confidence in treatment. The enablers and challenges included resource limitations, inter-professional group and individual participation, and pedagogy.

**Discussion:**

Future efforts in IPE mental healthcare practice can focus on garnering sustained institutional support, identifying and investing in committed faculty, encouraging greater learner participation, and making iterative changes to the IPE program structure to facilitate involvement of inter-professional disciplines for better patient care.

## Introduction

There has been increasing recognition of the benefits of interprofessional education (IPE) and practice in healthcare, not only for the learners [1, 2] but also patients [1, 3] and within the healthcare team and setting [4,5,6]. For example, patients felt more included in their partnerships with healthcare providers, and were able to receive personalized and consistent information from their healthcare team [3]. This increased patients’ knowledge of their condition and their confidence in managing it, which in turn gave them greater motivation to care for themselves within the endocrinology setting [3]. Patients also had improved clinical outcomes in the form of reduced mortality rates and shorter length of hospital stays within the intensive care and general ward settings [1]. The healthcare teams reported improved attitudes towards teamwork, lower clinical error rates within the emergency department [4], increased productivity, and reduced staff turnover as a result of increased job satisfaction within nursing homes [5]. In the mental healthcare setting, earlier studies and reviews of IPE interventions and practice within the setting of mental healthcare mainly focussed on outcomes which corresponded to Levels 1 and 2 of the Kirkpatrick evaluation model which included learner reaction and changes in knowledge, skills and perception [7,8,9]. For instance, learners were generally satisfied with the knowledge gained from the course [10,11,12]. They reported greater confidence in their ability to care for patients with complex conditions [13], increased self-awareness of their knowledge gaps [14], and better understanding of the value of teamwork [15, 16]. A recent review of such IPE interventions examined mainly the attitudes, and level of knowledge or skills acquisition of learners but not other changes within the healthcare setting or perspectives of patients or caregivers [17]. Reeves et al. [18] in an earlier review of IPE interventions across healthcare professions concluded there was a lack of studies on practice outcomes and there is a need to evaluate such outcomes across specific healthcare professions. Thus, whilst the benefits of inter-professional education (IPE) and practice have been reported in the literature for various clinical settings and patients within the healthcare professions, it is unclear whether the findings are generalizable to other settings and there is no recent review of specific practice outcomes as well as enablers and barriers to IPE effectiveness within the mental healthcare setting. A better understanding of the impact of such IPE interventions on outcomes related to healthcare setting and patients and caregivers would clarify beneficial elements of IPE interventions employed, as well as the effectiveness of IPE interventions on specific practice outcomes.

In light of the need to evaluate IPE intervention related practice outcomes and based on sparse extant reviews of such outcomes within mental healthcare, we thus sought to examine 1) the type of IPE interventions employed in mental healthcare settings using Strosahl’s typology framework [19], 2) practice outcomes related to healthcare setting and patients using Kirkpatrick’s model of training evaluation [8], and 3) enablers and challenges related to the effectiveness of IPE interventions in mental healthcare settings in order to guide the development of such future IPE programs.

## Methods

We conducted this scoping review in accordance with the methodology of the Joanna Briggs Institute for scoping reviews and used the five steps framework to guide the process [20, 21]. The first step involved identifying the main research questions of interest, which were as follows:

1. What were the modes of IPE intervention in mental healthcare based on Strosahl’s typology?
2. What were the outcomes of IPE interventions for mental health professionals based on the Kirkpatrick’s evaluation model with focus on outcomes within the healthcare setting and patients?
3. What were the enablers and challenges for the positive outcomes of IPE interventions in mental healthcare?

The second step involved identifying relevant studies for inclusion in the review. We searched several databases (including PubMed/Medline, Web of Science, Scopus) for relevant studies that examined IPE interventions for mental health professionals from database inception until December 2023. The search strategy used was as follows:

(((interprofession*[title/abstract]) OR (“inter-profession*”[title/abstract]) OR (multidisciplin*[title/abstract]) OR (“multi-disciplin*”[title/abstract]) OR (interdisciplin*[title/abstract]) OR (“inter-disciplin*”[title/abstract]) OR (transdisciplin*[title/abstract]) OR (“trans-disciplin*”[title/abstract]) OR (crossdisciplin*[title/abstract]) OR (“cross-disciplin*”[title/abstract])) AND (education[title/abstract]) AND ((“mental health”[title/abstract]) OR (psychiatr*[title/abstract])) AND ((outcomes[title/abstract]) OR (practice[title/abstract]) OR (learn*[title/abstract])))

**Figure 1:**
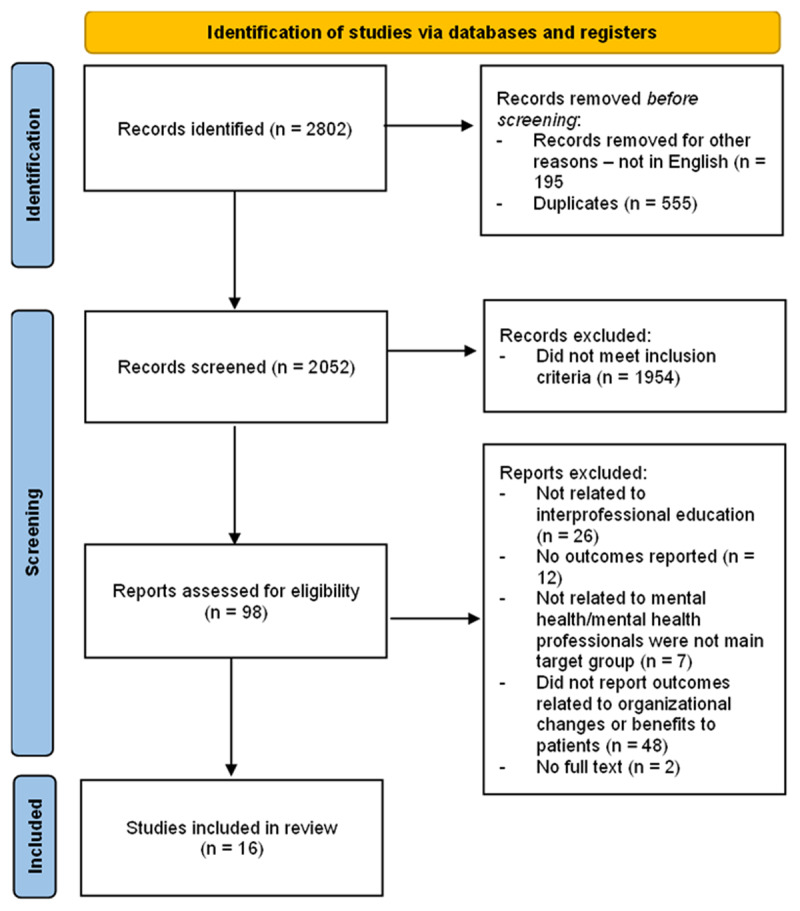
PRISMA Flowchart of included studies.

The third step involved study selection. Articles were included if it 1) involved IPE with mental health professionals as one of the main target groups, 2) reported outcomes of IPE interventions that included Level 4 practice outcomes in healthcare setting and practice using the Kirkpatrick’s model for training evaluation (see below), and 3) was published in English. Articles were excluded if 1) it did not involve IPE component as part of the intervention, 2) did not involve the mental healthcare setting. We manually screened the titles and abstracts of identified reports to determine whether they met the inclusion criteria, before proceeding to review full reports of promising studies. Two independent reviewers simultaneously screened the titles and abstracts. In case of any inconsistency between reviewers, the disagreement was resolved by a third reviewer. The PRISMA chart (see Figure 1) detailed the article selection process.

We then proceeded with the fourth step that involves charting the data. We extracted variables of interest, namely the mode of the IPE intervention, enablers, challenges, and outcomes. We applied a uniform approach to the extraction as far as possible, incorporating Strosahl’s typology for categorizing modes of IPE intervention [19], and the modified Kirkpatrick’s training evaluation model for categorizing reported outcomes [8]. There are five main categories of training methods described in Strosahl’s typology [19].

1. Didactic training involves methods of direct instruction such as workshops and modular courses [17, 19].
2. In vivo training involves job shadowing, academic detailing, and supervised clinical practice using core competencies framework with the aim of promoting awareness of different styles of practice across disciplines [17, 19].
3. Case vignettes and videos may involve role plays, case presentations, or best-practices video training sessions to teach learners how to apply their knowledge in the clinical context [17, 19].
4. Service manuals for clinical and administrative services are used to detail the policies and procedures, as well as the philosophy and parameters of services provided [17, 19].
5. Mentoring and extended practice consultation is used to identify and solve practice issues over time [17, 19].

The modified Kirkpatrick evaluation model based on work by Freeth and colleagues [8] describes four main levels of IPE intervention outcomes, with the second and fourth level divided into two subcategories. Detailed descriptions of each of these levels have been provided in Table 1. These subcategories are not mutually exclusive and one study may report outcomes pertaining to both levels 4a and 4b. We focused on Level 4 outcomes in this review related to healthcare setting and patients.

**Table 1:** Modified Kirkpatrick Evaluation Model.


LEVEL	THEME	DESCRIPTION

1	Reaction	Learners’ reactions to the interprofessional learning experience, and their views of working collaboratively in the course of learning

2a	Modification of attitudes/perceptions	Learners report changes in their attitudes or perceptions about interprofessional learning and working collaboratively. Changes in learners’ attitudes or perceptions towards other professional groups.

2b	Acquisition of knowledge/skills	Learners report increased knowledge about other professional groups, their respective roles in a team, and a better understanding of how to work together synergistically

3	Behavioural change	Learners demonstrate application of their interprofessional learning to their practice setting, including the way they work with their colleagues and patients/caregivers

4a	Change in organizational practice	Evidence of changes at the organizational level, work culture, and delivery of care as a result of learners’ application of interprofessional learning to their practice setting

4b	Benefits to patients/clients	Evidence of improvement in subjective or objective indicators of health or well-being of patients/clients/caregivers. Greater involvement and empowerment of patients/clients/caregivers in treatment process.


Finally, the fifth step involves collating, summarizing and reporting the results which were summarised below and in Table S1.

## Results

**Figure 2:**
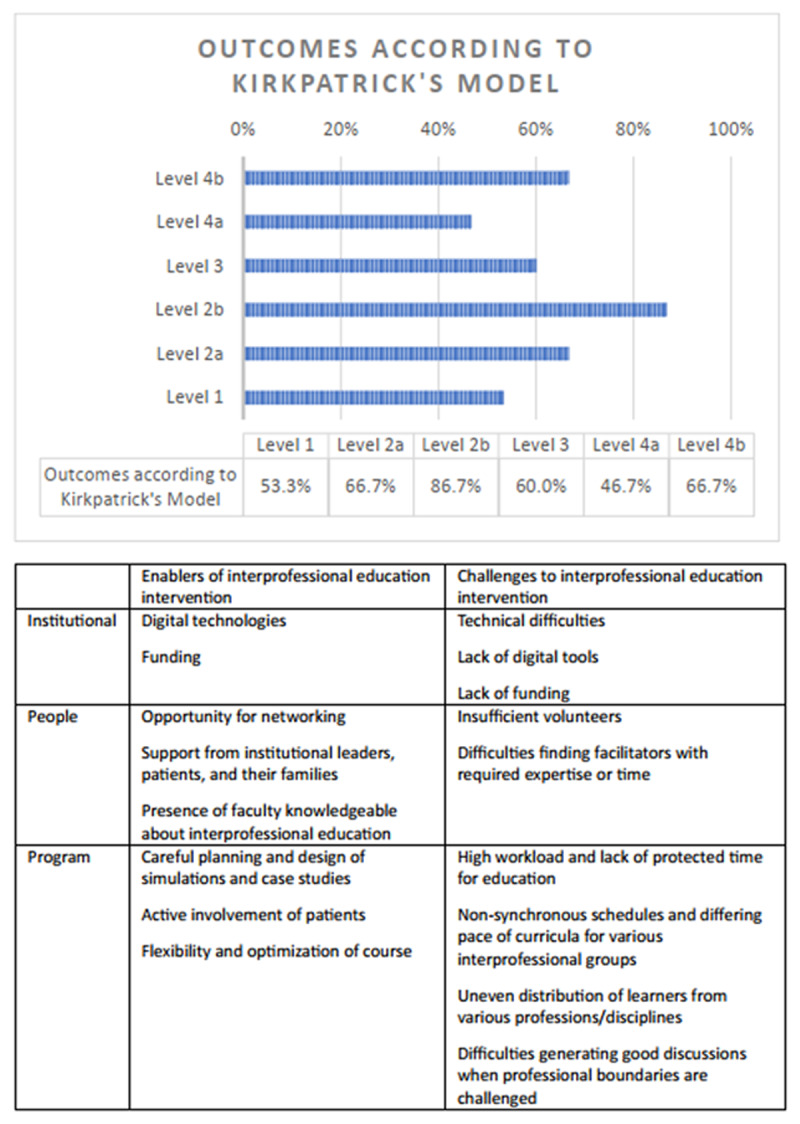
Summary of outcomes, enablers, and challenges of interprofessional education.

Out of 2052 papers identified and screened, 16 papers were included in this review. Half of the included studies were reported in the last decade and mostly conducted in the UK, USA, Europe, and Australia (see Figure 1 for PRISMA flowchart of screened and included studies and Table S1 for details of relevant studies). In terms of modes of IPE intervention according to Strosahl’s typology, the commonest mode was didactic teaching (10/16 studies, 62.5%), followed by in vivo training (8/16 studies, 50.0%), and case vignettes (4/16 studies, 25.0%). The duration of the IPE interventions varied from a few days to 24 months and occurred in a variety of healthcare settings such as community (7 studies, 43.8%), rural (4 studies, 25%), acute inpatient (3 studies, 18.8%), recovery-focussed and other contexts (2 studies, 12.4%). Figure 2 is a summary of the outcomes, enablers, and challenges of interprofessional education which are elaborated below.

### Outcomes related to healthcare setting and patients according to Kirkpatrick’s training evaluation model

Six studies (37.5%) reported on outcomes corresponding to level 4a (IPE intervention-related changes in practice within healthcare setting) according to the Kirkpatrick’s evaluation model of training evaluation [22,23,24,25,26,27]. This came in the form of shifts in service culture as other members of the multidisciplinary team followed the lead of clinicians that attended training and increased their engagement with family members [26]. Other changes included an increase in network connections, referrals, and collaborations with specific allied health services [22,23,24,25, 27].

Ten studies (62.5%) reported on outcomes corresponding to level 4b (IPE intervention-related changes from the patient’s perspective) of the Kirkpatrick’s training model of training evaluation [28,29,30,31,32,33,34,35,36,37]. Some of these outcomes were based on feedback given to the practitioners by their patients [29, 31, 33], while some were based on observations by the practitioner [29, 30, 32]. Others were based on improvements in clinical/objective measures [28, 30, 34,35,36] or self-report questionnaires [30, 34]. Patients saw improvements in clinical outcomes such as improvement of mental status [29, 34], depressive and anxiety symptoms, suicidal ideation [37], psychosocial functioning [28], reduced use of psychotropic medications, fewer restraints and more discharges into community-based facilities and programs [35, 36], and were more satisfied with their treatment [33, 34]. They also reported feeling empowered, valued and being listened to through their participation in the interdisciplinary partnership [29, 31], and could take initiative in organizing resources to support their well-being [28]. Patients were more willing to place their confidence in their care team [31] and collaborate with them [28]. Patients benefitted from not having to repeat their concerns and medical history multiple times when treated by an interprofessional team [32], and were able to provide a more holistic picture of their issues rather than tailored towards a certain professional group [32]. The benefits also extended to caregivers as they were able to learn more about the professionals involved in caring for their loved ones [31], and were given the opportunity to participate in planning of the care for their loved ones [30].

### Enablers of IPE intervention

Elements that facilitated the success of IPE can be categorized into three main groups, namely institutional, people and program level factors. Regarding institutional factors, external resources in the form of digital technologies facilitated the implementation and continuation of IPE [24, 29]. Building the necessary infrastructure for education was an important prerequisite for the provision of quality care inter-professionally [29, 37] and implementation of collaborative efforts [31]. Support in the form of funding encouraged greater participation from learners and contributed to the sustainability of IPE efforts [24]. Regarding people factors, the opportunity to connect with other professionals facilitated the exchange of ideas and experiences [32]. Attendance and commitment to learning were enhanced by support from leaders within and outside the organization, as well as patients and their families [31]. For teaching elements, the presence of faculty helped in provision of supervision [29, 37], stimulating critical thinking, troubleshooting problems that arose [29], and acting as role models for learners [22, 31]. Staff and near-peer teachers who were knowledgeable about IPE, the key subject matter, and able to appreciate its value were particularly important for creating an open learning climate and guiding learners [22, 24, 31]. With regard to program factors, careful planning and design of the simulations and case studies were crucial in ensuring fidelity [26], as well as smooth execution of the course logistically [29]. Patients were also invited to play an active role in some IPE courses [26, 31, 34] by sharing their experiences [31], facilitating and develop the training program [26], and being involved in the teamwork [34]. In terms of course structure and design, learners appreciated the use of active, experiential learning techniques and mediums such as role plays [22]. Flexibility and optimization of course duration, timing, and structure for the unique needs of learners were additional useful enablers of IPE interventions [22, 24, 25].

### Challenges to IPE intervention

Obstacles and barriers to the implementation or success of IPE could similarly be grouped into three main categories, namely institutional, people and program level factors. Regarding institutional factors, some programs cited the problem of having to work with limited resources and funding [28, 31]. A lack of digital tools and materials or technical difficulties hindered learner’s full participation in the programme [22, 23, 29]. For people factors, insufficient volunteers could limit the sustainability of some IPE interventions [37]. A number of studies also reported difficulties in finding facilitators with the required expertise or time [24, 31]. When the longevity and sustainability of professional networks and groups were in doubt, this had a negative effect on the willingness of inter-professional members to participate in such IPE interventions [24]. Regarding program factors, a high workload and lack of protected time for education limited participant’s ability to take time off to participate in training [31]. Non-synchronous calendars, schedules, and differing pace of curricula from various inter-professional groups were additional challenges for the implementation of IPE [33]. Distribution of professionals from various disciplines were not always even, and some groups were occasionally over-represented [25, 27, 31]. It was difficult to ensure that the content of the workshop was equally beneficial across disciplines as the expertise and depth of knowledge differed across members in some instances [22, 25, 27, 33]. Participants may struggle to generate good discussions and reflections or find their place in the group when their professional boundaries were challenged [32].

## Discussion

Overall, our scoping review revealed several findings concerning IPE intervention-related practice outcomes within the context of mental healthcare. First, the main healthcare setting outcomes pertained to shifts in practice culture, engagement with family members and increase in network connections and collaborations with other inter-professional groups. Second, the main reported patient outcomes related to clinical improvements, patient empowerment, satisfaction, confidence in treatment, and caregiver involvement. Third, the enablers and challenges related to physical and personnel resources, inter-professional group, individual participation, and pedagogical elements.

An earlier systematic review about IPE interventions across different disciplines about a decade ago noted a lack of studies that measured healthcare setting processes or patient outcomes [18]. In our current review of IPE interventions specific to mental healthcare, organizational level changes were mostly seen in terms of closer collaboration and increased connections amongst various healthcare services and inter-professional groups. One study reported a shift in culture of patient care across the healthcare team as a result of the influence of selected members who attended IPE training [26]. This suggested that a greater emphasis may be placed in the midst of IPE programs to encourage reflection and discussion about how such interventions can be translated to actual collaborative practice within the healthcare context [26]. This can be further deliberated within the community of practice when the members of the different inter-professional groups bring their learning points back to their teams so that the knowledge and insights can be shared [26, 28]. This sharing of learning points across community of learners and practice in their daily work can be useful to consider given the positive impact that such IPE interventions can foster in terms of greater collaborations and that members of inter-professional groups had reported difficulty with finding time to attend such sessions [10, 11, 13, 38]. It is imperative for organisational leadership to encourage staff to engage and participate in such IPE sessions by intentionally setting aside time and sustaining conversations around inter-professional collaborations to benefit patient care [24, 39, 40].

Regarding patient outcomes, patients benefitted from IPE interventions based on various clinical outcomes in mental healthcare related to reduction of symptomatology, suicidal ideation, changes in clinical management, psychotropic use, and improved functioning [28, 34,35,36,37]. Although it is less certain how long such gains in clinical outcomes would be sustained following the cessation of IPE sessions, the results provided evidence for the benefits of greater inter-professional collaboration and practice. In addition, patients subjectively reported being more satisfied [33, 34] and confident with their treatment and healthcare team [31]. They were empowered to be more actively involved in their treatment and to share their experiences with their treatment providers [29, 31], and this awareness of the importance of shared decision-making saw caregivers increasingly being regarded as part of the treatment process [30]. These findings demonstrated that the benefits of IPE interventions are not confined to learners alone, but can positively transform the quality of patient care in real world settings [1, 3]. This behoves greater attention to incorporate relevant elements of IPE and collaboration into the curriculum of health professions education over time [1].

In order to facilitate the successful incorporation of IPE into health professions education, studies have found enablers at the institutional (e.g., the provision of appropriate resources), people (e.g., social support), as well as program levels (e.g., thoughtful design of the teaching and course structure). With the uptake in adoption of digital technologies for health professions education as a result of the evolving COVID-19 pandemic, this has greatly improved the reach, sustainability, and cost-effectiveness of IPE programs [13, 24, 29, 41,42,43,44,45] without compromising its effectiveness [46]. As such, healthcare settings may want to tap on advancements in digital technology and infrastructure to support the continuity of inter-professional collaboration and practice for better clinical outcomes. Regular interactions within inter-professional teams during workplace learning had been shown to improve learner’s perceptions towards IPE and collaboration regardless of the type of activity [47]. Apart from digital infrastructure, the proper structuring of IPE sessions is equally crucial. Considerations need to be placed on content of course, support of faculty with the appropriate experience, expertise, and commitment to guide and supervise the learners [13, 41, 48]. Of note, instilling a sense of trust and belonging is essential for creating a more meaningful learning experience [13]. This can be facilitated through the use of small group teaching which provides a learning environment that promotes student engagement [49, 50].

Some barriers to IPE interventions identified in this review were observed in IPE sessions across disciplines at the institutional, people, and program levels [51]. Institutional level challenges included adequate sustained funding, allocation of protected time, and support [52, 53]. Challenges at the individual level included availability of committed, trained faculty who often had other clinical commitments [54], learner attitudes, and reactions to responses of others concerning their professional roles [55]. Facilitators, in particular, was a key aspect in the success of IPE interventions. Having a consistent facilitator [25] and opportunities to meet them face to face were crucial in establishing rapport for the participants [22]. This in turn promoted trust in the facilitator, and allowed participants to open up during the training sessions [22]. Good rapport with the facilitator also helped to mitigate any issues encountered with technology or resource constraints [22]. Facilitator’s characteristics such as being open and committed were also found to be key drivers of gaining participants’ interest and involvement [31], and served to empower the staff involved in the training process, while also being respectful of their vulnerability [31]. Facilitator’s knowledge of the subject matter and the networks or reputation they have established were important in establishing credibility and support for training interventions [31]. The professional networks that they have established also allowed them to reach out more effectively to the relevant participants within IPE interventions [31].

Program level challenges included appropriate pacing and scheduling across the different inter-professional groups [53]. A previous study examining barriers to IPE for medical and nursing students concluded that existing curricula needed to be malleable, and revised to address areas of practice overlaps for IPE to work effectively [56]. Other areas for sustainable implementation of IPE can include the commitment of the respective faculties involved in IPE [31], synchronization of existing curricula of each inter-professional group with respect to comparable content depth and learning aims [16], adequate preparation and training of lecturers for the IPE approach [57], and prioritizing the added value for patients and their caregivers in the teaching of IPE topics [31].

### Practical implications for IPE interventions in mental healthcare

There are several practical suggestions that are worthy of consideration arising from this review which may be applicable to healthcare contexts outside mental healthcare. First, it is important to have continual and sustained support and commitment of the institution towards IPE initiatives with impact on inter-professional collaboration and practice. This entails adequate and appropriate funding and support for time taken by staff involved in such programs [52, 53]. Second, there is a need to identify committed faculty who constitute a community of practice which champions IPE and collaboration efforts at the teaching, learning, and practice levels [31]. Faculty development is integral to enhance the skills within the core faculty group which can be expanded over time [57]. Third, for the learner, there is a need to encourage reflection about their professional identity, roles, challenges, and benefits of engaging in IPE and clinical care [58]. Creating a culture of psychological safety, respect for each other, encouragement of open sharing and personal feedback can foster a deeper sense of trust and honesty which can strengthen the interprofessional networks formed [13]. Fourth, in terms of program structure of IPE, intentional efforts are needed to work towards accommodating the different clinical workloads of the faculty and learners, finding suitable slots within the different training timetables to encourage maximal participation, and developing content that is relevant for the disciplines involved in the workplace setting [27, 39, 53]. Fifth, future research is warranted to examine the outcomes of such IPE interventions over a longer period of time with objective and subjective ratings. Other areas to be investigated include specific enablers or barriers towards IPE effectiveness across different inter-professional groups and cost-benefit analyses of such IPE interventions in real world settings.

There were several limitations in this study. First, there was still a modest number of studies which reported IPE intervention-related practice outcomes in mental healthcare. Second, the heterogeneity of duration of studies, modes of IPE interventions as well as treatment settings implied even smaller subsets and did not allow for appropriate study comparisons across these variables of interest. In addition, there was a lack of related studies in Asian countries which limited the ability to examine differences across geographic regions and cultures. One contributing factor could have been our use of studies published in English only as an inclusion criterion. Third, there is a dearth of formal ratings used. For example, the assessment of clinical outcomes such as symptomatology, psychosocial functioning, and treatment adherence can incorporate use of validated rating scales reported in the literature.

In conclusion, we found that more recent reports of IPE interventions in mental healthcare had observed positive outcomes within different healthcare settings (such as increased collaboration and referrals within practice networks), patients (including improvements in symptomatology, clinical management, functioning, satisfaction and confidence in treatment teams) and caregivers (such as greater involvement and shared planning). With the enablers and challenges of IPE programs viewed in the context of mental healthcare, future efforts can focus on garnering sustained institutional commitment and support, identifying committed faculty and investment in faculty development, encouraging greater learner participation, reflection and feedback, and making iterative changes to the IPE program structure to facilitate involvement of all stakeholders across inter-professional disciplines for the betterment of clinical care of patients irrespective of the healthcare contexts.

## Additional Files

The additional files for this article can be found as follows:

10.5334/pme.1084.s1 Supplementary file 1. Summary of data from all included studies.

10.5334/pme.1084.s2 Supplementary file 2. Preferred Reporting Items for Systematic reviews and Meta-Analyses extension for Scoping Reviews (PRISMA-ScR) Checklist.
