# Supplementary material for: Inter-Professional Education Interventions, and Practice Outcomes Related to Healthcare Setting and Patients Within Mental Healthcare: A Scoping Review
Source: Perspect Med Educ. 2024 Feb 20;13(1):108–18. doi: 10.5334/pme.1084 (PMC10885830; doi:10.5334/pme.1084)
Supplement: Supplementary file 1. — Summary of data from all included studies. [file pme-13-1-1084-s1.pdf]

No.	Author (Year)<br><br>Country	Mode of IPE intervention (Strosahl's typology)	Enablers of outcomes	Challenges of outcomes	Practice Outcomes	Outcome Level (Kirkpatrick's Evaluation Model)
1	Zhou et al. (2022) [37]<br><br>USA	[Didactic training, In vivo supervised clinical practice]<br><br>10-12 sessions over ~12 weeks<br><br>Education (for volunteering attending physicians, psychiatry residents/fellows, psychology interns, social workers, and medical students)<br><br>• Student-run clinic for LGBTQ+ population<br>• All volunteers received instruction in LGBTQ+ affirming care<br>• At the start of rotation, all post-graduate year 2s received didactic instruction on principles in supportive psychotherapy<br>• Each resident was paired with an attending psychiatrist or psychologist for weekly individual psychotherapy supervision for the	Adjustments to post-graduate year 2 psychiatry resident responsibilities due to COVID-19 allowed for opportunity to create a mandatory rotation with the student-run clinic<br><br>Building infrastructure for provider education and supervision at multiple levels has enabled the clinic to provide quality mental health care<br><br>Ability to provide multiple treatment modalities contributed to positive clinical outcomes	Lack of volunteers as clinic expands<br><br>Free service model may not be sustainable in the long run	<u>Level 2b</u><br>Trained medical students to lead a dialectical behavioural therapy-based Coping Skills group – valuable learning opportunity and expanded psychotherapy service offerings<br><br><u>Level 4b</u><br>Better engagement of patients<br><br>Patients experienced a significant 5.1-point reduction in their PHQ-9 scores and a 6.7-point reduction in their GAD-7 scores after 12 weeks of treatment<br><br>Patients experienced a significant reduction in frequency of suicidal ideation	2b<br>4b

		duration of the rotation				
2	Browne et al. (2021) [29]<br><br>USA	[Clinical case vignettes, In vivo supervised clinical practice]<br><br>14 weeks of learning activities<br><br>Education (for psychiatric mental health nurse practitioners, pharmacy students, and social work students)<br><br>• Interprofessional team telehealth with rural patients<br>• Students assigned to small interprofessional teams and underwent two virtual standardized patient simulations<br>• Students also engaged in team telehealth visits with patients from two rural clinics<br>• At all stages, students were supervised by clinical faculty members from nursing and social work with expertise in mental health	Meticulous logistical planning for course execution<br>• Interprofessional team met for more than a year before launching online class<br>• Provided students with didactic technology training<br>• Started the class with simulated patients to familiarize students with technology before working with actual patients<br>• There was a project staff/faculty member standing by during all class sessions to troubleshoot any technical difficulties	Issues with student internet connectivity<br><br>Inexperience with telehealth technology	<u>Level 1</u><br>Felt that course was beneficial to their training<br><br><u>Level 2a</u><br>Increased readiness to work in teams and deliver team-based care to patients<br><br>Better prepared to provide telehealth interventions<br><br>Learned about the potential of telehealth in practice<br><br>Improved rural clinic telehealth readiness<br><br><u>Level 2b</u><br>Learned about each other's roles and responsibilities in an interprofessional team<br><br>Increased ability to teach other students about their scopes of practice<br><br>Better understanding of rural health needs<br><br><u>Level 4b</u><br>Patients reported improved mental health status<br><br>Patients felt empowered through	1<br>2a<br>2b<br>4b

					their participation in this academic-community partnership	
3	Higgins et al. (2020) [26]<br><br>Ireland	[In vivo supervised clinical practice]<br><br>8 sessions x 1.5 hours/week<br><br>Education (for mental health practitioners)<br><br>• Involved nurses, social workers, occupational therapists, and psychiatrists<br>• Co-facilitated group psychoeducation intervention to improve recovery promoting beliefs and practices of the staff and organization<br>• Jointly facilitated by a servicer user/family member, and a clinician from the mental health service	Combines experiential knowledge of peers (service users and family members) and the expertise of clinicians in the development of the programme<br><br>Engaged practitioners in real concrete examples of recovery-oriented practices as opposed to decoupling recovery education from practice	One of the reasons many new initiatives fail to be sustained is that the focus is often on first-order change, as opposed to targeting the less tangible individual and organizational assumptions and thinking	<u>Level 1</u><br>Learners felt that the programme helped them to reconnect to the contextual realities of people's lived experience<br><br><u>Level 2a</u><br>Strengthened empathy and motivated them to deliver best standard of care, adopting more inclusive approaches towards families<br><br>Gained confidence in working and interacting with service users in a collaborative way, decreased risk adversity<br><br>Programme provided a space for deconstruction of assumptions<br><br>Encouraged reflection on own practice, question paternalistic care practices, and advocate upwards for change<br><br><u>Level 2b</u><br>Gained insight into and renewed understanding of both service users' and family members' experiences<br>• Reduced fear and anxiety	1<br>2a<br>2b<br>3<br>4a

					around family involvement<br>• Self-efficacy enhanced<br><br><u>Level 3</u><br>Participants began to speak about their learning to colleagues<br><br><u>Level 4a</u><br>In observing that participation in the programme increased engagement of clinicians with family members, other members of the multidisciplinary team followed suit, resulting in a subtle shift in service culture<br><br>Programme afforded participants their first opportunity to build a relationship of partnership with service users and family members<br><br>Programme generated further conversation about how to integrate perspectives of service users and families	
4	Marcussen et al. (2020) [34]<br><br><br>Denmark	[In vivo supervised clinical practice, Didactic training]<br><br>One weekly group tutorial + morning and evening shifts<br><br>Education (for students from health professions)<br><br>• Involved students from medicine, nursing,	Patients played an active role in the teamwork	Although there was improved patient satisfaction, the effect was smaller than expected and its clinical significance is difficult to ascertain<br>• Improvement was likely driven by patients' active	<u>Level 4b</u><br>Patients in the interprofessional training unit had better mental health status based on Short Form Health Survey (SF-36) and the Kessler Psychological Distress Scale (K10)<br><br>Patients in the interprofessional training unit reported higher satisfaction with their treatment	4b

		pedagogy, physiotherapy, and social work<br>• Interprofessional clinical training unit established in a psychiatric ward<br>• Students in the ward would participate in team-based care<br>• Students also met once a week for interprofessional group tutorials<br>• There was a comparison group in this study where students received traditional uniprofessional training during their placement		participation in their own care	based on Client Satisfaction Questionnaire (CSQ-8)	
5	Jentoft (2020) [32]<br><br>Norway	[Didactic training, In vivo supervised clinical practice]<br><br>>19 hours of learning activities<br><br>Education (for students from medicine, occupational therapy, and physical therapy)<br><br>• Mental health outpatient setting with elderly people living at home<br>• Interprofessional collaboration among	Process of collaborative writing was an important contribution to interprofessional learning in professional language and concepts<br>• Strengthened their confidence in professional and interprofessional capabilities<br>• Enabled them to reach common ground	Professional boundaries were challenged<br>• Home environment was not conducive for physiotherapists to conduct full examination<br>• Felt that more time was needed for preparation, supervision, and discussion<br><br>Students and the	<u>Level 2a</u><br>Collaborative environment encouraged learners to hold back and not interfere with each other/the client, allowing the client to concentrate on the whole group<br><br><u>Level 2b</u><br>Collaboration was effective and gave the assessment and intervention planning higher quality<br>• Developed better understanding of each professional's beliefs, values, knowledge, and actions	2a<br>2b<br>3<br>4b

		students which consisted of a seminar, two home visits, two health record writing sessions, and a final seminar		interprofessional supervisor found it difficult to have good discussions and reflections when students' professional boundaries were challenged<br><br>Occupational therapists struggled to find their professional role when other members of the interprofessional team focused on client's healthcare needs<br><br>Felt they had to hold back certain questions due to time limitations during the home visit<br><br>Felt discomfort about working in a group and observed by different professions, clinicians, and educators	• Helped to establish a more effective client-centred practice<br><br>Level 3<br>Participants learned to prioritize more time for the collaborative writing of the health record after their initial experience<br><br>Level 4b<br>Interprofessional collaboration led to less burden for the client as they did not need to repeat the same story or participate in several separate assessments<br><br>Client's story tended to be more holistic rather than geared towards a certain interest of the profession	
6	Westbury et al. (2018) [35]	[Didactic training, in vivo training]	Prescribing information was obtained from	Sub-optimal participation of prescribers (only 51%	Level 4b<br>13% reduction in mean prevalence of antipsychotic agent	4b

	Australia	1-hour educational session<br><br>Education (for staff working in residential aged care facilities)<br><br>• Comprised psychotropic medication audit and feedback, staff education, and interdisciplinary case review at baseline and 3 months, with final audit at 6 months<br>• One-hour educational sessions at baseline and 3 months	pharmacy packing programs rather than it being reported by staff (potential reporting bias) or by auditing medication charts (places demands on nurses' time)<br>• Feasibility of this study resulted in all 150 residential aged care facilities completing the intervention<br><br>Strategies built upon each other, starting with awareness raising by dissemination of local prescribing data, reinforced by staff education, and followed by interdisciplinary review	of those invited participated in academic detailing)	use over 6 months<br><br>21% reduction in mean prevalence of benzodiazepine use over 6 months<br><br>12% decrease in mean chlorpromazine dose per resident per day<br><br>23% reduction in mean diazepam dose per resident per day	
7	Phillips et al. (2016) [27]<br><br>Australia	[Didactic training]<br><br>6 hours of workshops<br><br>Education (for practitioners from various health professions)		Distribution of professionals from various disciplines were not always even – some groups were overrepresented at certain workshops	Level 2b<br>Improvements were reported in knowledge of assessment of chronic and psychological illnesses, management planning, relapse prevention, and patient perspectives	2a<br>2b<br>3<br>4a

		• Involved GPs, nurses, and mental health professionals<br>• A national interprofessional educational program addressing the care of patients with comorbid psychological and chronic physical illnesses<br>• The module was delivered in one 6-hour workshop, or two 3-hour workshops<br>• Facilitated by a local clinician with expertise in psychological care		Content of the workshop was sometimes not meaningful for specific health disciplines<br><br>No significant changes in the size of professional networks either overall or for each type of relation (information exchange, referral, or collaboration)<br><br>No significant differences in total number of network ties, directionality of information and referral flow, or frequency of interaction within networks<br><br>Some psychologists expressed concern that the rapid overview of complex psychological interventions might cause other participants to be over-confident about	Improvement in confidence to recognise patients with comorbid physical and psychological illnesses, use psycho-educational strategies, and meeting needs of carers<br><br><u>Level 3</u><br>Participants reported nearly 100% use of strategies taught at this workshop at baseline and 3-month follow up<br>• The most significant change in uptake of strategies was the GP group – increase in reported use of mindfulness and motivational interviewing<br><br>Intentions to change in areas relating to:<br>• Improved care planning and shared management, including better collaboration with local services<br>• Involving patients and carers more in disease management planning<br><br>Significant increase in mean frequency of interactions with psychologists<br><br><u>Level 4a</u><br>Increase in total number of network connections to four	

				their ability to deliver complex psychological interventions<br><br>Simply placing health professionals in proximity to one another in an educational session is not sufficient to generate changes in attitude or collaborative practice, and may even cement prejudicial attitudes towards each other	specific disciplines (exercise physiologists, psychologists, psychiatrists, and the Veterans and Veterans Families Counselling Service)<br><br>Decrease in use of non-specified counsellors in favour of increased links with specific psychological services (psychiatrists, psychologists, and the Veterans and Veteran Families Counselling Service)<br>• Shows greater awareness of the role and utility of specific psychological services	
8	Heath et al. (2015) [25]<br><br>Canada	[Didactic training, Clinical case vignettes]<br><br>10 sessions over ~20 weeks<br><br>Education (for individuals from all sectors of work who might benefit from a rural mental health training program)<br><br>• Involved nurses, social workers, community development specialists, youth regional coordinators, school counsellors, police officers,	Longer than most rural mental health training programs (multiple sessions over approximately 20 weeks)<br>• Allowed sufficient time for participants to form interprofessional relationships<br><br>Consistent facilitator	Low attendance at sessions for some professions (e.g paramedics, police, justice)<br>• Likely due to them choosing to attend sessions most applicable to them<br><br>No significant differences in attitude or perception change scores between different	<u>Level 1</u><br>Viewed the program as a reminder of their belief in the efficacy of interprofessional practice<br><br><u>Level 2a</u><br>Significant increase in positive attitudes towards interprofessional mental health care<br><br><u>Level 2b</u><br>Over 90% of respondents felt that program had positive impact on their understanding of teamwork in interprofessional mental health care	1<br>2a<br>2b<br>4a

		occupational therapists, dieticians, and physicians<br>• Rural mental health interprofessional training program<br>• Training comprised both didactic evidence-based mental health intervention material and interactive experiential teaching tools		communities, professions, ages, or years of experience<br><br>No significant positive change in perception of interprofessional collaboration<br>• Already high prior to the intervention<br>• Reflects pre-existing motivation and capacity in rural communities to practice collaboratively	Helped them develop better understanding of their own role (82.6% in agreement) and other professions' roles (91.3% in agreement) in collaborative mental health care<br><br>Reminded them about core principles of collaborative practice<br>• Especially the need to take time to plan their collaborations so that all appropriate people have been included and given ample opportunity to make a contribution<br><br><u>Level 4a</u><br>Helped them develop new knowledge, insights, and referral networks for interprofessional mental health practice<br>• While there had been good intra-institution collaborations, there was now more contact across sectors<br>• Learned what other professionals could do and had an opportunity to educate others about their areas of expertise as well<br>• Personal connection was critical for some participants	

					– helped them feel more comfortable contacting other professionals in the future	
9	Fletcher et al. (2014) [24]<br><br>Australia	[Didactic training, case vignettes]<br><br>1 session workshop<br><br>Education (for healthcare professionals in primary care and mental health care)<br><br>• Involved GPs, psychiatrists, paediatricians, psychologists, social workers, occupational therapists, and mental health nurses<br>• Interprofessional workshop initiated by the Mental Health Professionals Network<br>• Involves discussion of a case study of a client with a mental disorder, and a discussion of the possibility of generating an ongoing local network	Provision of guidance from the Mental Health Professionals' network was a key enabler of network success<br>• Particularly assistance in administrative tasks<br>• Provision of practical resources such as an online portal that raises awareness of network meetings<br><br>Funding for time spent coordinating and administrative support were key incentives for network participants to act as coordinators<br><br>Graded, flexible, and supportive approach<br><br>Identifying barriers and enablers, and modifying elements	Staffing levels limited the Mental Health Professionals Network<br><br>Mental Health Professionals Network's own lack of clarity about how best to support ongoing networks<br><br>Inability to provide networks with assurances about the longevity of the Mental Health Professionals Network, which jeopardized potential network likelihood of engagement<br><br>Occupational therapists and psychiatrists were somewhat less positive than other professional groups about the degree to which the workshops met their learning objectives	Level 1<br>Participants gave facilitators a mean rating of between 8.2 and 8.8 in six key areas (group management, knowledge, respect for all professions, time keeping, equity of input, clarity of instruction)<br><br>Workshop materials were also rated positively (mean ratings from 7 to 8)<br><br>More than 50% of the participants were very satisfied with the mix of professionals attending the workshop<br><br>Facilitators were positive about the support they received from the Mental Health Professionals Network and the project officers<br><br>Facilitators felt that their role was well-explained, the resources were valuable, and the structure of the workshop sessions was well organized<br><br>Between 90 to 95% of participants had their learning needs partially or entirely met with respect to:	1<br>2a<br>2b<br>3<br>4a

			of the project accordingly<br><br>Initiative was delivered at a time when many mental health professionals in primary care were eager and ready for opportunities for interdisciplinary networking	Psychiatrists were less likely than other professional groups to show interest in ongoing networking<br><br>Primary reason for the uncertainty about wanting to be part of an ongoing interdisciplinary network<br>• Majority (61%) said that they had not yet found a network that they would like to be part of<br>• 34% said it was too much effort<br>• 5% said they were already engaged in interdisciplinary networking<br><br>Social workers and mental health nurses were the most likely to take up an invitation to attend a network meeting while paediatricians and GPs were the least likely to do so	• Recognising the expertise of other mental health professionals<br>• Identifying referral pathways to other local mental health professionals<br>• Identifying opportunities for ongoing professional development and mutual support with other mental health professionals<br>• The participants' individual learning needs<br><br>Over 95% of participants indicated that workshops were partially or entirely relevant, and partially or entirely useful<br><br><u>Level 2a</u><br>98.8% of participants felt that interdisciplinary networking was important or very important post-workshop<br><br>52.9% felt that the workshops had very much increased their desire to engage in collaborative mental health care<br><br><u>Level 2b</u><br>Over 90% of participants indicated that their knowledge of other professionals' contribution to mental health care had increased a little or very much as	

					a result of attending the workshops<br><br><u>Level 3</u><br>When participants were asked explicitly whether they wanted to participate in an ongoing interdisciplinary network, over 70% indicated that they did<br><br>At the 14-week follow-up survey, 53% indicated that the workshop was moderately responsible for increases to their networking activities while 8% indicated that the workshop was extremely responsible<br><br><u>Level 4a</u><br>More than half also agreed that the workshops had very much assisted in creating ongoing local interdisciplinary network activity<br><br>Online resources helped participants to stay in contact with local mental health professionals for the purposes of consultation and referral of consumers<br><br>Online portal assisted mental health professionals to participate in local interdisciplinary networks by:<br>• Helping them learn about	

					other professionals in their area (60%)<br>• Expanding their networks (53%)<br>• Enabling them to organize events and network meetings (40%)<br>• Enabling them to RSVP to such events (47%)<br><br>81.2% of workshops on the Mental Health Professionals Network workshop list progressed to generate ongoing networks at a national level<br>• 36% of networks have met at least once<br>• In 48% of cases, psychologists have become network coordinates<br><br>Important to have strong coordinator and clear purpose for the network	
10	Curran et al. (2012) [23]<br><br>Canada	[Didactic training]<br><br>2-month psychiatry rotation, 1-day IPE workshop, 10 modules post-licensure program<br><br>Education (undergraduate students from various healthcare disciplines)<br><br>• Involves students from		Technical issues and signal delay<br><br>Interprofessional collaboration workshop<br>• No significant differences in attitudes towards interprofessional health care	Collaborative Mental Health Practice Interprofessional Module<br>• 98% agreed or strongly agreed that it has enhanced their understanding of interprofessional teamwork (Level 2b)<br>• 90% agreed or strongly agreed that it has enhanced their understanding of the mental health subject area (Level 2b)	1<br>2a<br>2b<br>3<br>4a

		medicine, nursing, pharmacy, social work, and clinical psychology		teams<br>• No significant differences in perception of effective interprofessional teams<br>• Could be due to ceiling effect<br><br>Rural mental health interprofessional training program<br>• No significant change in perceptions of collaboration in mental health care<br>• Could be due to ceiling effect<br><br>Lack of perceived change in self-assessed teamwork abilities may be attributable to the difficulty that trainees and health practitioners often experience in identifying their own weaknesses and the tendency to overestimate one's abilities	• 97% agreed or strongly agreed that it was a meaningful learning experience (Level 1)<br><br>Interprofessional collaboration workshop<br>• 90% agreed or strongly agreed that it enhanced understanding of interprofessional collaboration (Level 2b)<br>• 85% agreed or strongly agreed that they would recommend the workshop to others (Level 1)<br>• Participants did report practice changes in team meetings and rounds, and being more aware of professional roles with regard to patient referrals (Level 3)<br><br>Rural mental health interprofessional training program<br>• Highest rating was given for satisfaction with "opportunity for interaction provided" (Level 1)<br>• Significant improvement in attitudes toward interprofessional collaboration in mental health care (Level 2a)	

					• Increase in interprofessional referrals, inter-agency linkages, and collaborations (Level 4a)	
11	Kinnair et al. (2012) [33]<br><br>UK	[In vivo supervised clinical practice]<br><br>3 days of learning activities<br><br>Education (for undergraduate health professions students)<br><br>• Involved students from medicine, social work, mental health nursing, occupational therapy, midwifery, and pharmacy<br>• Practice-based interprofessional education<br>• Students would assess mental health patients, analyse and reflect on their profession-specific learning related to mental health, and local interagency resources, and produce new solutions for care		Timing of the learning event within each profession's curriculum<br>• Nursing students in Phase I were final year students who had specialist mental health knowledge<br>• They felt like they had little to learn from other students and were less motivated to engage<br>• This was remedied by moving the learning earlier within the nursing curriculum	Phase I<br>• Medical and social work students increased their perceived knowledge (Level 2b) while mental health nurses did not<br>• Nurses had already learnt the content previously<br>• Patients valued interprofessional team-based care (Level 4b)<br><br>Phase II<br>• Students appreciated how their profession contributed to team working (Level 2a)<br>• Valued the process of working and learning in a mixed student team (Level 1)<br>• All students considered future interprofessional working as the way to improve the quality of patient care (Level 3)<br>• Social work students emphasized the value of gaining deeper insights into the theory and practice of mental health team working (Level 2a)<br>• Preferred interactive activities and did not want to	1<br>2a<br>2b<br>3<br>4b

					listen to local experts<br><br>Phase III<br>• Nursing students considered how they would work in mental health teams in the future, and paid more attention to listening to other professionals' opinions that might differ and using other skills (<u>Level 2a, 3</u>)<br>• Social work students had a more sophisticated understanding of the interprofessional approach (<u>Level 2b</u>)<br>• The occupational therapists, pharmacists, and midwives valued understanding more of other professionals' roles within mental health teams and who to refer their patients to (<u>Level 2b</u>)	
12	Wizner (2010) [36]<br><br><br>USA	[Cases]<br><br>Education (for law students, psychiatric residents, and hospital social workers)<br><br>• Three examples of how Dr. Howard Zonana collaborated with Yale Law School's clinics in the teaching of forensic psychiatry<br>• Collaborate to develop			The Donaldson Project<br>• Panels concluded that more than half of the patients were inappropriately confined to locked wards in the hospital<br>• Served to focus attention on the need for periodic review of committed patients to determine who among them should no longer be confined under current legal and psychiatric standards	2b<br>4a<br>4b

		a project to review the cases of long-term involuntary patients at the hospital to identify those who legally can no longer be involuntarily confined under the *Donaldson* criteria over 20 weeks			• The law students, psychiatric residents, and hospital social workers who participated in this project gained knowledge, practical judgment, and professional skills through their work together (Level 2b)<br>• By the end of the first 6 months of meetings, every member of the plaintiff class was participating in a day program<br>• Majority of plaintiff class were leaving the hospital grounds to participate in sheltered workshops, supported employment, or social and recreational programs<br>• There were on-grounds program at the hospital as well where they were taught daily living and self-help skills and engagement in recreational activities in the community<br>• On the hospital wards, treatment had improved, with all members having their medications reduced or terminated (Level 4b)<br>• The use of restraints and PRN medication fell (Level 4b)<br>• Hospital staff were energized and professionalized by the	

					attention that was paid to their work by the commissioner at the weekly meetings and by the advocates from the law school coming onto the wards to learn about the services being provided (<u>Level 4a</u>)<br>• In slightly over two years of interdisciplinary case conferences, all members of the plaintiff class had been discharged from the hospital to small community-based residences and day programs (<u>Level 4b</u>)<br><br>Case of African woman refugee<br>• Judge found client's claim for asylum credible despite her inconsistent testimony and lack of emotional expressiveness<br>• The forensic fellow in charge of this case, together with the cooperation and support of the law students, was able to persuade the client to accept treatment<br>• The law students, whom the client had grown to trust, managed to accompany the client to weekly therapy and waited for her outside until she was able to attend	

					therapy on her own<br>• Dr. Zonana played a vital role in guiding the legal advocates in understanding how to be effective in a nonadversary setting through collaboration with professionals from other disciplines (Level 2b, 4a)<br>• Dr. Zonana helped the law students read and understand hospital records and made them aware of the institutional and bureaucratic complexities involved in service delivery and systemic change (Level 2b, 4a)	
13	Furness et al. (2011) [31]<br><br>UK	[In vivo training]<br><br>Education (for practitioners and students from 13 professions in health and social care)<br><br>• Included eight varied practice settings<br>• The first pilot site was in an acute mental health network of a large teaching hospital<br>• Facilitators (a nurse and an occupational therapist) developed a series of interprofessional problem-based learning sessions for students	Facilitators complemented one another professionally and were well known within the facility<br>• Important in establishing credibility of and support for the project amongst practitioners<br>• Teamwork allowed facilitators to consider ideas together<br><br>Facilitators' knowledge of the site	One pilot site's facilitators were unable to involve medical professionals and felt that time limitation was the main barrier<br><br>Despite facilitators' considerable efforts, service users with greater cognitive and communicative impairments were less able to participate<br><br>Challenging to locate resources (especially staff time) to	Level 1<br>Project was relevant and practitioners believed it would benefit staff, students, and service users<br><br>Students felt that the group discussions were purposeful and involved everybody<br><br>Level 2a<br>Participants were optimistic about the potential for new ways of thinking to improve collaboration<br><br>Level 2b<br>Practitioners felt that they had greater insight into the input of different professional groups	1<br>2a<br>2b<br>3<br>4b

		and newly qualified staff<br>• The second pilot site took place within a community-based organization offering support to adults with learning disabilities<br>• Two facilitators with links to the organization developed a series of workshops that brought professionals and students from a range of disciplines together with service users and parents	and pre-existing lines of communication had facilitated access to practitioners and service users, and accelerated progress<br><br>Pilot sites were already committed to interprofessional working and were therefore fertile grounds for the implementation of this project<br><br>Support was given by all parties involved, including service users and their caregivers<br><br>Facilitators' characteristics (openness and flexibility) and commitment (time and effort, research and enthusiasm) had been key drivers to gaining people's interest and involvement, especially given practitioners' heavy workload	facilitate ongoing work	Students' understanding of other professionals' roles had grown<br><br>Learners had increased confidence, conflict management, and team working skills<br><br><u>Level 3</u><br>The experiential learning helped students to bridge the theory-practice gap in their training<br><br><u>Level 4a</u><br>Practitioners were inspired to increase interprofessional collaboration<br><br>Staff-patient communication saw improvement<br><br><u>Level 4b</u><br>Caregivers learned more about professionals involved in the care of their loved ones<br><br>Service users gained confidence in the professionals and reported feeling valued and listened to<br><br>Service user involvement was crucial to participants' positive perceptions<br>• Students valued speaking to carers and hearing service users share their experience	

			The enthusiasm of a medic at one of the pilot sites had increased the project's appeal to medical practitioners<br><br>Sensitivity of service user inclusion<br>• Facilitators allowed service users to take ownership of the project<br>• Empowered and respected service users at all stages of the process<br>• Were mindful of the potential vulnerability of service users as well		• Service users appreciated the opportunity to share their experiences and advocate for their ability to make choices	
14	Church et al. (2010) [22]<br><br><br>Canada	[Clinical case vignettes]<br><br>8 x 2-hour video sessions with 2 full-day onsite training, and case-based discussions over a 4-month period<br><br>Education (for all professions that may need professional development in	Time set aside<br>• Create space for purposeful conversation<br>• Spaced out programme structure allows participants to reflect on the material between	Designing a curriculum that is relevant to all participants<br><br>Participants gave lowest satisfaction scores for technical factors (signal delay)<br>• Relationship with the facilitator	Participants gave highest ratings for their satisfaction with "opportunity for interaction"<br><br><u>Level 2b</u><br>Significant increase in their confidence in dealing with mental health issues and in using different interventions<br><br>Programme had sparked the	2b<br>3<br>4a

		the area of mental health)<br><br>• Involved community development workers, dietetics, family medicine, nursing, nurse practitioners, occupational therapy, police/justice, school counselling/psychology, social work, and youth workers<br>• Interprofessional mental health continuing education programme for those in rural areas<br>• Delivered largely via distance technology<br>• Case-based small-group exercises over a 4-month period	sessions<br><br>Trust in the facilitator<br>• Shared knowledge freely, listened carefully, and responded positively<br>• Established personal connections with the participants<br>• Clinical experience gave her credibility<br>• Shared failures as well as successes<br>• Modelled the kind of relationship with participants that she was teaching in the sessions<br><br>Experiential format<br>• Opportunity to role play with colleagues in a non-threatening environment<br>• Trust in facilitator was a factor in encouraging	helped to mitigate the effects of the technology	participants' desire for more mental health training<br>• Fuelled by seeing the benefits to their work<br><br><u>Level 3</u><br>Developed a more reflective practice<br>• Re-assess approach to mental health<br>• More aware of own attitudes and beliefs around mental health care<br>• Sensitised them to possible mental health issues with their clients<br>• Thought back to what had been discussed in the sessions post-programme<br>• Was an opportunity to re-evaluate their priorities and develop a new perspective on their work<br><br>Integrated new knowledge and skills into their practices<br>• Applied it not only to mental health practice but also to other aspects of work<br>• Learned to listen more to clients before jumping in with advice<br><br><u>Level 4a</u><br>Increased their referral network	

			participants to open up<br><br>Community coordinator as bridge<br>• Community coordinators provided a link between the facilitator and the participants<br>• They were able to identify which professionals would benefit, encouraged them to attend, and maintained contact with participants throughout<br><br>Participant background and experience in mental health<br>• Those who already had some relevant experience benefitted the most, attended the most consistently, and were more likely to apply it to		New mental health collaborations had emerged<br>• Occurred primarily in the communities which had the highest levels of attendance	

			their practice<br><br>More face-to-face meetings were desired by participants<br>• Initial onsite meetings were critical in establishing a rapport with the facilitator			
15	Barnes et al. (2006) [28]<br><br><br>UK	[Didactic training]<br><br>1 day/week over 2 years<br><br>Education (for mental health professionals)<br><br>• Involved mental health nursing, social work, occupational therapy, psychologists, and psychiatrists<br>• 2-year, part-time, postqualifying course		Barriers to implementation of learning were often about lack of resources<br><br>Some concerns were expressed about tokenism and representation<br><br>Problems at first timepoint of evaluation:<br>• Students felt that they could not criticise service users' views in the way they might challenge professionals, were afraid to ask questions	<u>Level 1</u><br>Students valued hearing first-hand experiences of mental illness from service users<br><br><u>Level 2a</u><br>Improvement in attitudes towards partnership with service users<br>• Awareness of the imbalance of power between service users and professionals made students more conscious of sharing decision-making and a needs-led approach<br><br>Other team colleagues welcomed the new skills which students brought back to their teams from the programme<br><br><u>Level 2b</u><br>More practical knowledge gained about resources and advocating	1<br>2a<br>2b<br>3<br>4b

				• Students were critical of the teaching skills of some service users<br>• Service-user trainers were not always given the respect of other lecturers<br><br>Solution to problems identified at first timepoint:<br>• Train service users in presentation skills<br>• Introduced joint teaching sessions pairing a service user with an experienced teaching staff member<br><br>Problems with service users as students:<br>• Other students experienced difficulty debating issues freely in front of classmates who were service	for service users<br><br><u>Level 3</u><br>Students explored empowerment and user involvement in their treatment<br><br><u>Level 4b</u><br>Service users had changed their demands<br>• Collaborating to change problems instead of fighting the system<br><br>At the start, students took the lead in setting up/supporting user groups, but over time, users were taking more initiative and students took on a more supportive role instead<br><br>Improvement in social functioning and life satisfaction for users in the programme group based on the Life Skills Profile (LSP) and Life Satisfaction Scale (LSS) were significantly greater than those in the comparators	

				users<br>• When service users became unwell, programme staff were unprepared to handle it<br>• Service users were unable to apply their skills to clinical practice and the assignments had to be modified for them<br>• None of the service-user students qualified successfully at the end of the programme because they failed to submit assignments or dropped out due to ill mental health		
16	Carpenter et al. (2006) [30]<br><br>UK	[Didactic training]<br><br>1 day/week over 2 years<br><br>Education (for mental health professionals)			Level 4b<br>Almost all users believed that students treated them with respect and understood their experience of mental ill health<br><br>Three-quarters of users felt that	4b

		See Barnes et al. (2006) for details of the programme			students had worked with other agencies to ensure that their needs were met<br><br>Over three quarters of users stated that students had involved them in care planning as much as they wished<br><br>Significantly greater proportions of users in the intervention group had been asked by students whether they wanted a carer or family member involved in planning their care<br>• Attributable to programme's emphasis on care planning and family intervention	
